# A Comparative Study of Color Stability and Fluoride Release from Glass Ionomer Cements Combined with Chlorhexidine

**DOI:** 10.5005/jp-journals-10005-1181

**Published:** 2013-04-26

**Authors:** AR Prabhakar, Kirti Pattanshetti, S Sugandhan

**Affiliations:** Professor and Head, Department of Pedodontics and Preventive Dentistry, Bapuji Dental College and Hospital, Davangere-577004 Karnataka, India; Senior Lecturer, Department of Pedodontics and Preventive Dentistry Dr DY Patil Dental College and Hospital, Nerul, Navi Mumbai Maharashtra, India; Professor, Department of Pedodontics and Preventive Dentistry, Bapuji Dental College and Hospital, Davangere, Karnataka, India

**Keywords:** Resin-modified glass ionomer cement, Chlorhexidine diacetate, Fluoride release

## Abstract

**Background:** Restoring carious teeth is one of the major treatment needs of young children. Glass ionomer cement (GIC) systems had become the most important dental restorative and luting materials for use in preschoolers, children and teenagers. Several attempts in developing GIC with antibacterial effects by addition of bactericides, such as chlorhexidine, have been reported.

**Aim:** Aim of the study was to evaluate and compare the color and fluoride ion release of conventional and resin-modified GICs in combination with 1.25 and 2.5% chlorhexidine diacetate.

**Materials and methods:** The control groups consisted of conventional GIC and resin-modified GIC. The experimental groups consisted of conventional and resin-modified GIC groups, consisting of 1.25 and 2.5% chlorhexidine. A total of six groups were included with each group being allotted 20 specimens for the evaluation of color stability and 10 specimens each were allotted for the evaluation of fluoride release.

Color and fluoride release were recorded using spectrophoto-meter and fluoride selective electrode respectively at 24 hours 7 days and 1 month.

**Results:** Resin-modified GIC groups showed less color stability and better fluoride release at the end of the study compared to conventional GIC groups.

**Conclusion:** There was no significant change in color and fluoride release between 1.25 and 2.5% conventional GIC and also between 1.25 and 2.5% resin-modified GIC combined with chlorhexidine diacetate at the end of the study. Conventional GIC showed better color stability and less fluoride release compared to resin-modified GIC.

**How to cite this article:** Prabhakar AR, Pattanshetti K, Sugandhan S. A Comparative Study of Color Stability and Fluoride Release from Glass Ionomer Cements Combined with Chlorhexidine. Int J Clin Pediatr Dent 2013;6(1):26-29.

## INTRODUCTION

The mutans group of streptococci is strongly associated with the initiation of dental caries. Different measures have been developed in order to eliminate mutans streptococci, such as plaque control by means of professional tooth cleaning followed by fluoride applications, dental flossing, supervised tooth brushing with fluoride toothpaste or self-administered oral hygiene programs, in different combinations. Treatment with chemical antimicrobial agents has been extensively studied using various administration modes, such as dentifrices, rinsings, chewing gums, adherent pastes, lozenges, application of gels or varnishes and depot devices.^1^

Due to the high frequency of recurrent caries after restorative treatment, much attention has been paid to the therapeutic effects revealed by direct filling materials. Remineralization by the release of fluoride is a representative, but the antibacterial effect is another important property because inactivation of bacteria means a direct strategy to eradicate the cause of dental caries^2^

Dental restorative materials also have an antibacterial effect. Glass ionomer cements (GICs) inhibit the growth of mutans streptococci and some antibacterial activity of such material may be due to fluoride release. The low pH in the vicinity of freshly mixed cements and release of cations are the other important antibacterial properties of these materials. Jedrychowski et al increased the antibacterial activity of GIC by addition of chlorhexidine (CHX) without compromising the mechanical properties of the material.^1^ CHX has been shown to be the most suitable agent in reducing mutans streptococci due to its increased susceptibility when compared to other microorganisms. Since, CHX is retained in oral structures from which it is slowly released, this is one reason that its antimicrobial effect is significantly longer than other agents.^3^

Considering the above concepts, the present study was undertaken to evaluate and compare the color stability and release of fluoride of conventional and resin-modified GICs in combination with 1.25 and 2.5% CHX diacetate.

## MATERIALS AND METHODS

CHX diacetate (Kemcolour International, Ankleshwar, Gujarat, India) which is commercially available as solid substance was added to GC Fuji II (GC Corporation, Tokyo, Japan) and GC Fuji IILC (GC Corporation, Tokyo, Japan) in order to obtain 1.25, 2.5% concentrations of CHX in the GIC formulation. To obtain 1.25 and 2.5% diacetate formulations, 0.22 and 0.44 gm CHX diacetate were mixed with each 15 gm of GC Fuji II and GC Fuji IILC respectively. GIC-CHX mixture and GIC liquid was manipulated according to the manufacturer's instructions at room temperature on a mixing pad with a plastic spatula and placed in a brass mold of diameter 1.5 cm and height 2 mm. The cement was compressed between two mylar strips, sandwiched between two glass slabs and held under constant hand pressure until the cement was set. For resinmodified GIC, the material was light cured for 40 seconds on each side with the light of wavelength of 450 to 490 nm (Bee Cool, plus top light LED curing). Then each specimen was polished with composite polishing kit. Individual specimens were placed in airtight labeled plastic containers containing 25 ml of distilled water.

Twenty specimens of each 1.25 and 2.5% CHX combined with conventional and resin-modified GICs were used for evaluation of color and 10 specimens of each 1.25 and 2.5% CHX diacetate combined with conventional and resin-modified GICs were used for evaluation of fluoride release.

Specimens were divided into two groups as follows

1. *Group I:* Control group
  *Group IA:* Conventional GIC
  *Group IB:* Resin-modified GIC
2. *Group II:* Test group
  *Group IIA:* Conventional GIC + 1.25% CHX diacetate powder
  *Group IIB:* Conventional GIC + 2.5% CHX diacetate powder
  *Group IIC:* Resin-modified GIC + 1.25% CHX diacetate powder
  *Group IID:* Resin-modified GIC + 2.5% CHX diacetate powder

Color measurements were made using Minolta Spectrophotometer (CM-330 ld) with a 10 mm aperture and D65 illuminant for the time period of 24 hours, 7 days and 1 month for each specimen of control and test groups. The spectrophotometer was calibrated using its specified calibration plate provided by the manufacturer before each series of measurement. Individual specimen was placed on the aperture and the base line reading ΔE (total color change) was recorded as displayed on the computer.

The color change (ΔE) between time intervals was calculated using the equation:

ΔE = √(ΔL*)^2^ + (Δa*)^2^ + (Δb*)^2^

Fluoride concentration was determined using Orion ion specific fluoride electrode (mode 94-09, 720 A) for the time period of 24 hours, 7 days and 1 month for each specimen of control and test groups. Fluoride in the distilled water was determined with the addition of total ionic strength adjustment buffer (TISAB) decomplexing agent. The groups tested are presented in results.

## RESULTS

### Color Stability

Intergroup Comparison

There was no significant change in color observed between the control and test groups of conventional GICs and the same was observed for even resin-modified GICs. But there was significant change in color observed between control and test groups of conventional when compared to resin-modified GICs at the end of the study (Table 1).

**Table 1:** **Table 1:** Intergroup comparison of the mean and standard deviation, the significance (p) value of the total color change (AE) between groups I and II and their subgroups at 24 hours, 7 days

*Groups*		*At 24 hours*		*At 7 days*		*At 1 month*
IA-IB		<0.01		<0.001		<0.001
IA-IIA		0.86 (NS)		0.99 (NS)		0.79 (NS)
IA-IIB		0.98 (NS)		1.00 (NS)		1.00 (NS)
IIA-IIB		0.99 (NS)		0.99 (NS)		0.80 (NS)
IB-IIC		0.97 (NS)		0.58 (NS)		0.99 (NS)
IB-IID		1.00 (NS)		0.99 (NS)		0.98 (NS)
IIC-IID		0.91 (NS)		0.79 (NS)		1.00 (NS)
IIA-IIC		<0.05		<0.001		<0.001

NS: Not significant

Intragroup Comparison

**Fig. 1:**
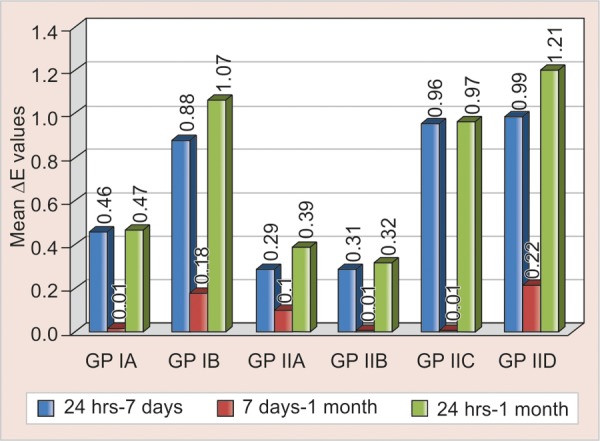
Intragroup comparison of change in color in control and test groups

There was highly significant (p < 0.001) change in color observed between 24 hours and 7 days (p < 0.001) and no significant color change between 7 days and 1 month in all the groups. At the end of the study, there was significant (p < 0.01) change in color was observed in all the groups (Fig. 1).

### Fluoride Release

Intergroup Comparison

There was nonsignificant decrease in fluoride release in test groups of conventional GIC compared to control group at the end of the study. There was highly significant decrease in fluoride release in test groups of resin-modified GIC compared to control group till the end of the study. There was increase in the fluoride ion release in both control and test groups of resin-modified GICs which was highly significant (p < 0.001) compared to control and test groups of conventional GIC at the end of the study (Table 2)

**Table 2:** **Table 2:** Intergroup comparison of the mean and standard deviation, the significance (p) value in fluoride release between groups I and II and their subgroups at 24 hours, 7 days and 1 month

*Groups*		*At 24 hours*		*At 7 days*		*At 1 month*
IA-IB		<0.001		<0.001		<0.001
IA-IIA		1.00 (NS)		0.97 (NS)		0.99 (NS)
IA-IIB		0.98 (NS)		0.85 (NS)		0.94 (NS)
IIA-IIB		1.00 (NS)		0.99 (NS)		0.99 (NS)
IB-IIC		0.97 (NS)		<0.05		<0.001
IB-IID		<0.01		<0.01		<0.001
IIC-IID		<0.05		0.89 (NS)		0.39 (NS)
IIA-IIC		<0.001		<0.001		<0.001
IIB-IID		<0.01		<0.001		<0.01

NS: Not significant

Intragroup Comparison

**Fig. 2:**
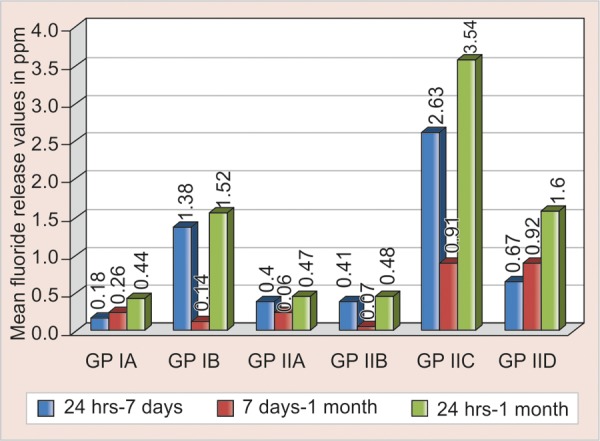
Intragroup comparison of fluoride release in control and test groups

There was highly significant (p < 0.001) decrease in fluoride release in GP IIA and GP IIB; significant decrease (p < 0.01) in GP IIC and GP IB and no significant decrease in GP IA and GP IID between 24 hours to 7 days. There was highly significant (p < 0.001) decrease in fluoride release in GP IA; significant decrease (p < 0.01) in GP IIA, GP IIB, GP IIC, GP IID and no significant decrease in GP IB between 7 days to 1 month. There was highly significant decrease (p < 0.001) in fluoride release GP IA, GP IIA, GP IIB, GPIIC and significant decrease (p < 0.01) in GP IID and GP IB (Fig. 2).

## DISCUSSION

As early as 1977, it was suggested that GICs could offer particular advantages as restorative materials in the primary dentition because of their ability to release fluoride and to adhere to dental hard tissues. They have therefore been suggested as the materials of choice for the restoration of carious primary teeth and also because they require short time to fill the cavity, GICs present an additional advantage when treating young children.^4^

Apart from the inherent antibacterial properties of the estorative material, different methods have been employed o further reduce or eliminate microorganisms underneath the estoration by adding antimicrobial agents, among them are he applications of cavity disinfectants^5^ or addition of CHX o GIC.^6^ Clinical trials have shown that CHX reduces *. mutans* count which is the prime organism causing dental aries in relation to those of other bacteria in plaque and aliva.^7^

### Color Stability

In the present study, both the control and test groups of conventional GIC showed significant change in color in the specimens stored for 1 month compared to the specimens stored for 24 hours which was taken as baseline values. Largest color change occurred from 24 hours to 7 days and 24 hours to 1 month. Results from 7 days to 1 month showed that there was no significant change in color, the results of the control group corroborates with the previous study done by Imparato et al (2007).^8^ Resin-modified GIC showed significant change in color in the specimens stored for 1 month compared to the specimens stored for 24 hours which was taken as baseline values. Largest color change occurred from 24 hours to 7 days and 24 hours to 1 month, the results of the control group is consistent with the previous study conducted by Yap et al (2001).^9^ Results from 7 days to 1 month showed that there was significant change in color in the control and 2.5% test groups and no significant change in color in 1.25% test group, the results of control group corroborates with the previous study done by Imparato et al (2007).^8^ Both the control and test groups of resin-modified GIC showed significant color change compared to control and test groups of conventional GIC at the end of 1 month. The results of the control groups were observed similarly in the previous studies.^8^

In our study, the control and test groups of conventional GIC presented better color stability than the control and test groups of resin-modified GIC, the results of the control groups corroborates with the previous *in vitro* study done by Imparato et al (2007).^8^ According to Yap et al (1999), resin-modified GIC undergoes color changes during light polymerization. This color change may be attributed to the photopolymerization of the resin components as the acid-base reaction is retarded. The delayed acid-base reaction in addition to water sorption by the resin components may result in post-polymerization color changes. The potential for color change may also exists for increased body discolorations, surface staining because of their hydrophilic monomers and incomplete polymerization, physical adsorption or physicochemical reactions in the material, wear or chemical degradation can increase the susceptibility of the material to extrinsic staining.^9^

### Fluoride Release

Test groups of conventional GIC showed increased release of fluoride in the first 24 hours and decreased release at 7 days. However, the control group showed no significant decrease in fluoride ion release from 24 hours to 7 days which is in agreement with previous studies by Forsten (1977).^10^ At the end of 1 month, there was significant decrease in fluoride ion release in control and test groups of conventional GIC, results of control group is consistent with previous studies conducted by de Araujo et al (1996), Yap et al (1999).^11,12^

The test and the control groups of resin-modified GIC showed increased release of fluoride in the first 24 hours and decreased release at 7 days. The results of control group are in consistent with previous studies.^13^ However, there was no significant decrease in 2.5% group between 24 hours and 7 days. At the end of 1 month, there was significant decrease in fluoride release in test and control groups, the results of control group are in agreement with the previous studies conducted by de Araujo et al (1996) and Yap et al (1999).^11,12^

Both the control and test groups of resin-modified GIC showed increased fluoride release than the control and test groups of conventional GIC till the end of the study, the comparison of the control groups is in agreement with previous studies^14^

Within the limitations of this study, incorporation of CHX diacetate in concentrations of 1.25 and 2.5% to conventional GIC did not significantly changed its color and fluoride release compared to conventional GIC alone, but there was decreased fluoride release in both the test groups of resin-modified GIC compared to resin-modified GIC alone. At the end of the study, both the control and test groups of resin-modified GIC showed less color stability and better fluoride release compared to control and test groups of conventional GIC.
